# RA-PLA: Retrieval-augmented graph convolutional networks for protein-ligand binding affinity prediction

**DOI:** 10.1371/journal.pone.0357278

**Published:** 2026-09-22

**Authors:** Karim Abbasi, Hossein Banadkuki, Reza Sepahvand, Shima Rashidi

**Affiliations:** 1 Mosaheb Institute for Mathematical Research, Kharazmi University, Tehran, Iran; 2 Computer Engineering and Information Technology Department, University of Qom, Qom, Iran; 3 Department of Artificial Intelligence and Data Science, College of Science and Technology, University of Human Development, Sulaymaniyah, Kurdistan Region of Iraq; National University of Singapore, SINGAPORE

## Abstract

Protein-ligand binding affinity (PLA) prediction is essential for computational drug discovery, yet existing deep learning methods typically employ a single globally trained model that fails to adapt to individual query samples. In this paper, we propose a novel end-to-end framework that retrieves hard protein-ligand pairs—samples whose nearest neighbors have conflicting labels under manifold smoothness constraints—and integrates them via a semi-supervised graph convolutional network (GCN). For each query, we automatically construct a graph where nodes represent protein-ligand pairs and edges encode pairwise similarities. Our model jointly learns joint descriptors, graph topology, and the GCN predictor. During inference, we fine-tune the model per query using its retrieved hard neighbors.We evaluate on four benchmarks: PDBbind, Davis, KIBA, and BindingDB. Our method consistently outperforms state-of-the-art approaches. On Davis, we achieve a Concordance Index (CI) of 0.952 ± 0.005 (1.6% improvement over NerLTR-DTA) and AUPR of 0.815 ± 0.005 (7.6% improvement). On KIBA, we achieve CI of 0.916 ± 0.002 and AUPR of 0.871 ± 0.005, outperforming DeepCDA by 2.7% and 5.9%, respectively. On PDBbind, our CI of 0.894 ± 0.002 represents an 11.4% improvement over PLA-MoRe. On BindingDB, we achieve AUPR of 0.518 ± 0.009 (5.9% improvement over DeepCDA). In cold-target generalization across four unseen protein families, we achieve average AUPR improvements of 15.5%. Paired t-tests confirm statistical significance (p < 0.05), and ablation studies validate that both hard sample retrieval and end-to-end learning are essential for performance gains. Our framework demonstrates that query-specific adaptation significantly enhances both accuracy and generalization in PLA prediction.

## 1. Introduction

Protein-ligand binding affinity (PLA) prediction is a crucial step in the computational drug discovery process [1–3]. The primary goal of PLA is to determine the binding affinity between a drug (ligand) candidate and a protein sequence [4,5]. PLA measurement through experiments is expensive and time-consuming. Hence, computational-based methods receive more attention [6–8]. To date, numerous approaches have been proposed in this research area. Computational approaches generally consist of two steps: feature extraction and task prediction. In the feature extraction step, discriminative features are extracted from the raw input. Feature extraction methods are divided into data-driven and non-data-driven methods [9–12]. In data-driven methods, features are learned automatically, while in non-data-driven approaches, features are extracted manually (hand-crafted features). The task prediction step takes the features as input and then maps them to the interaction space.

In classic PLA prediction approaches, features are usually extracted through non-data-driven methods like extended-connectivity fingerprints (ECFP) [13], rapid overlay of chemical structures (ROCS) [14], molecular linear notation by circular traverse (MLNCT), and fragment-based descriptors [15,16]. Many hand-crafted feature extraction algorithms are available [3,17]; hence, one of the main challenges in this area is determining which feature extraction algorithms are appropriate for the specific task.

In recent years, deep learning-based models have received more attention and improved the performance of PLA prediction [18–20]. In most deep learning-based approaches, first, a feature encoder for drug molecules and a feature encoder for protein sequences are trained. Then, these feature vectors are fused to construct a feature representation for the protein-ligand pair, which is then fed into the MLP to predict the affinity value. In other words, a single learned model can be used to predict the affinity value for all unseen protein-ligand pairs. We believe that the generalizability of this approach is limited. For each unseen protein-ligand pair, some training protein-ligand pairs might have more information than others. To incorporate this knowledge, we have designed a model that retrieves some training protein-ligand pairs for each unseen protein-ligand pair and uses those pairs to learn a specific model for each unseen pair. It means that each test sample has its prediction model, which helps the model improve its performance.

To incorporate the knowledge of the retrieved training samples in inferring the label of the query sample, we have utilized a semi-supervised learning method. The reason is that all of the retrieved samples are labeled training samples, and the query sample is unlabeled (semi-supervised setting). We have chosen to utilize semi-supervised GCN as the learning method to infer the label of the query sample. In this case, the nodes of the graph correspond to the feature vectors of the protein-ligand pairs, and the edges between the nodes represent the similarity between protein-ligand pairs. Based on this setting, to infer the label of the query sample, GCN leverages the knowledge of neighborhood protein-ligand pairs (i.e., similar pairs), which aligns with the paper's motivation.

To design a model with high generalization ability, we develop a robust task prediction network model by integrating hard similar samples into the test sample. Hard protein-ligand pairs are retrieved and integrated into interaction affinity prediction using a semi-supervised graph convolutional network for each test sample. To determine hard samples, the similarity of the query sample is computed with all training samples and then sorted. In the sorted vector, a sample is labeled as hard if its nearest neighbors have differing class labels. It should be noted that the class labels refer to the active/inactive classes.

To recap, the proposed model is a unified end-to-end framework with three specialized sub-networks: 1) Descriptor Sub-network: learns joint representations for protein-ligand pairs, 2) Adjacency Sub-network: learns pairwise similarities to construct the graph topology, and 3) GCN Prediction Sub-network: propagates information through the graph to predict test sample affinity. This work is the first to demonstrate that modeling the graph topology between test samples and their hard training neighbors improves prediction performance. All components are optimized jointly during end-to-end training.

The paper is structured as follows: Section 2 reviews related work on affinity prediction and graph-based learning. Section 3 details the datasets, while Section 4 introduces the proposed unified framework. Finally, Section 5 evaluates the model through benchmarks and ablation studies, and Section 6 discusses results, limitations, and future directions.

## 2. Related work

In this section, the recent advances in PLA prediction are reviewed. Ozturk et al.[21] utilize a CNN to extract features for both compound and protein. Abbasi et al. [17] introduced an approach that utilizes CNN, LSTM, and a two-sided attention mechanism to extract relevant features. Also, they employed domain adaptation techniques to learn more about discriminant features. These approaches use fully connected layers as prediction layers. Some methods utilize auxiliary knowledge, such as protein-protein interaction (PPI) graphs, to design a more accurate PLA prediction model [22–24]. Wang et al. [25] applied the random walk with restart method to calculate the topological similarity matrices using the PPI network, gene ontology, and drug fingerprint, as well as drug side effects. They utilized a multimodal deep autoencoder (MDA) to integrate multiple similarities and learn high-level features, which were then fed into a multi-layered neural network to predict ligand and protein interactions. Lim et al. [26] introduced a distance-aware graph attention algorithm to extract graph features of intermolecular interactions from 3D structural information. The learned features are fed into the fully connected layers to predict interaction affinity between the ligand and protein target. Ru et al. [27] introduced a method that utilized a ranking framework to predict affinity values, called NerLTR-DTA. They used learning-to-rank (LTR) algorithms to learn the model. Their approach provides the priority order of proteins related to the query drug and the priority order of ligands to the target protein. Zhou et al. [7] proposed a multimodal drug-target interaction prediction model called MultiDTI. Their method integrates the heterogeneous network into drug-target interaction by mapping it into a common space. This is done by integrating drug similarity, target similarity, side effects, and disease into the loss function. Shim et al. [28] used the similarity of drug-drug and protein-protein interactions to provide a 2D input to feed into the 2D convolutional layer. The corresponding columns in the drug-drug and protein-protein similarity matrix are taken for a drug-protein pair, and its outer product is computed. Yuan et al. [22] concentrated on feature aggregation and introduced a FusionDTA approach. They introduced a novel multi-head linear attention mechanism to aggregate global information. Aleb [29] has introduced two multilevel attention-based models for PLA prediction. Nguyen et al. [30] introduced a method in which a drug is represented as a graph. Then, it is fed into a graph convolution network (GCN), graph attention network (GAT), graph isomorphism Network (GIN), and GAT-GCN to learn the representation of the drug molecule. To represent the protein sequence, they used a 1D-CNN. They have shown that representing drugs as graphs could improve performance. Son and Kim [31] introduced a method that utilized a graph convolutional network called GraphBAR. In their approach, the ligand-protein complex is fed as input to GCN. Each graph has multiple adjacency matrices, which are created using different distance metrics. Liu et al. [32] introduced a new molecular representation in which ligand-protein complexes are modeled as Dowker complexes. Then, combinatorial Laplacian matrices are constructed by generating at different scales and passed into the Riemann zeta functions to generate molecular descriptors. Nguyen and Wei [33] introduced a novel algebraic graph learning score (AGL-Score) model to generate low-dimensional representations from physical and biological information. Li et al. [34] introduced a protein-ligand binding affinity prediction model called PLAMoRe. They proposed a new representation of learning that utilizes both structural knowledge and bioactive properties. Autoencoders are used to learn bioactive feature encoders from multisource information, including chemical, target, network, cellular, and clinical data. Wang et al. [35] introduced a new deep learning-based protein–ligand binding affinity prediction approach named DeepDTAF. In their approach, the protein-binding pocket is first used as input to extract the local feature, which is then combined with the global context feature. They have used a dilated convolutional network to extract long-range interactions.

## 3. Datasets

In this section, datasets are explained. To evaluate the proposed approach, four well-known datasets, including PDBbind [36], Davis [37,38], KIBA [39,40], and BindingDB [41], are used. The following sections provide detailed explanations of each dataset.

**PDBbind**. We have utilized PDBbind v2016, which contains three sets: the general set, refined set, and core set. The general set is filtered to select better-quality ligand-protein complexes, called the refined set. In this dataset, proteins are recorded in pdb format, and ligand structure data are recorded in mol2 or sdf format. We have chosen training, validation, and test sets that are consistent with other methods to ensure a fair comparison. Therefore, the core set is used as a test set [42].

***Davis.*** This dataset comprises the interactions of 442 unique proteins (from the kinase protein family) and 68 unique ligands. The respective dissociation constant (K_d_) value measures the binding affinity of protein-ligand interaction. The K_d_ measure is transformed into log space (𝑝K_𝑑_), similar to [17,37], as follows:


$$\begin{array}{c} {pK}_{d}=-{\text{log}}_{\text{1}\text{0}}\left(\frac{K_{d}}{{\text{1}\text{0}}^{\text{9}}}\right) \end{array}$$ (1)

***KIBA.*** This dataset includes 246,088 interactions between 467 unique proteins and 52,498 unique ligands. This dataset is filtered, so proteins or ligands with fewer than ten interactions are removed. This dataset measures binding affinity using the KIBA score, which combines kinase inhibitor bioactivities from various sources, including IC50, Ki, and Kd [43].

***BindingDB.*** It is a public dataset that provides the binding affinity of each pair, stated using at least one measure, such as IC_50_, EC_50_, K_i_, and K_d_. In this study, the K_i_-labelled samples, including 234491 pairs, are used. To evaluate the generalizability of the approach, similar to [17,44], we exclude the four classes of proteins entirely, including nuclear estrogen receptors (516 samples), ion channels (8,101 samples), receptor tyrosine kinases (3,355 samples), and G-protein-coupled receptors (77,994 samples) from the training set. The remaining protein-ligand pairs are divided into the training set with 101,134 samples and the test set with 43,391 samples.

## 4. Method

**Fig 1:**
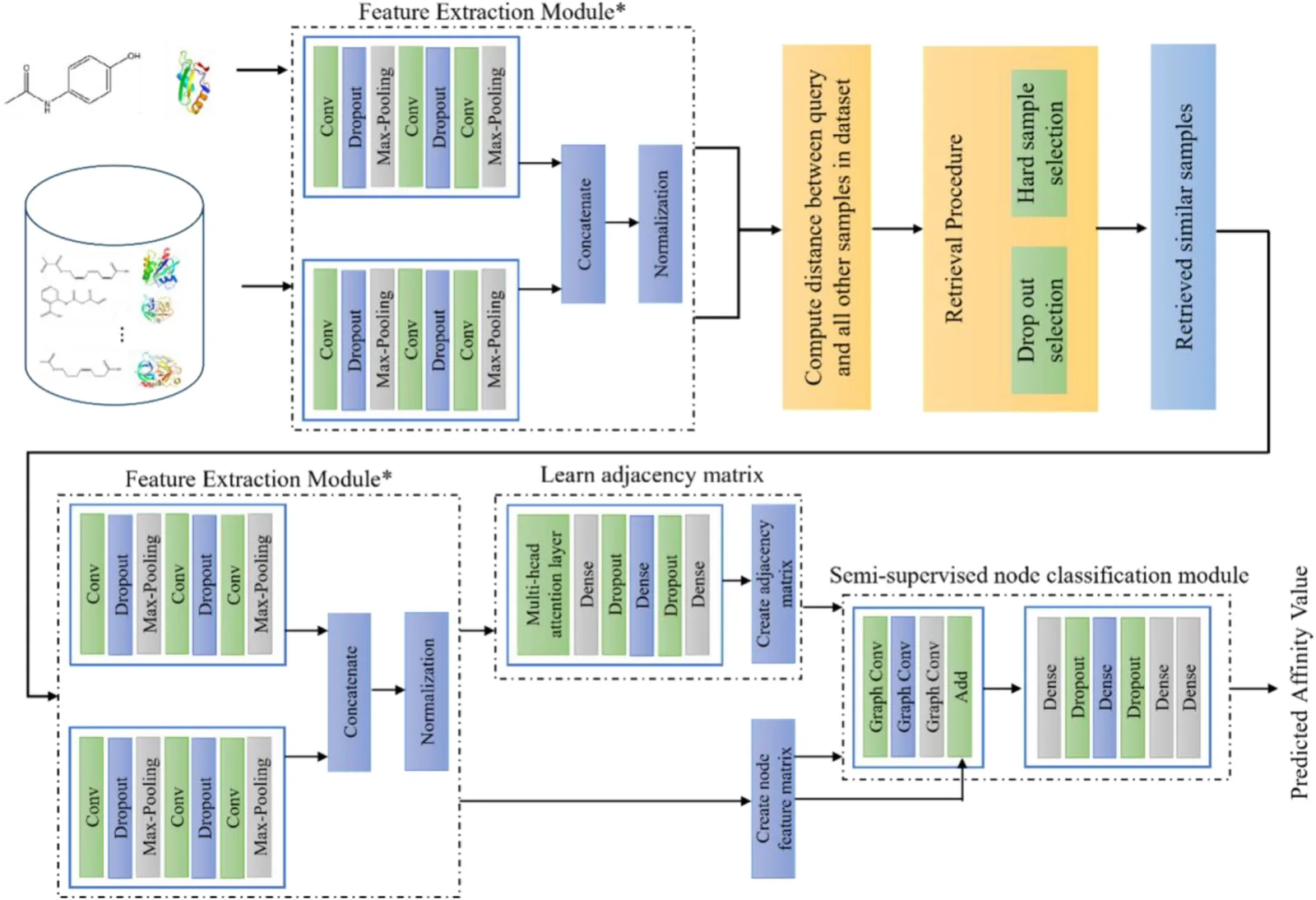
The overall schematic of the proposed approach using Strategy 3 (hard sample retrieval with dropout). First, hard samples (those with conflicting neighbor labels) are retrieved for the query, combined with 50% random dropout selection. Then, the query sample and all retrieved samples are fed into the model to predict the affinity value. It should be noted that * means that the feature extraction module is shared between the retrieval procedure and the forward pass of the model.

In this section, the proposed model is explained in detail. The overall schematic of the proposed approach is presented in Fig 1. The following section begins with a presentation of the problem formulation. Then, feature extraction and task prediction steps are explained.

### 4.1. Problem formulation

Let *c* and *p,* respectively, denote a drug compound (ligand) and a 1D protein sequence of amino acids. All training samples are shown by $D={\left\{\left(c^{i},p^{i}\right),y^{i}\right\}}_{i=\text{1}}^{N}$ where *N* denotes the number of training samples; it should be noted that each sample is a protein-ligand pair. Also, $y^{i}$ shows the strength of binding, which can be either a binary binding affinity (active or inactive) or a quantitative binding affinity (e.g., IC_50_, K_d_, K_i_). In the proposed approach, both cases are considered.

In the test step, a test sample is shown by $\left(c^{test},p^{test}\right)$ is fed into the proposed model. To this end, in the first step, a set of hard training samples for the test sample is retrieved, as shown by $S={\left\{(c^{j},p^{j})\right\}}_{j=\text{1}}^{\left|S\right|}$, where |S| indicates the number of retrieved similar training samples. It should be noted that the number of retrieved samples might differ for each input query sample (see section 4.2). The main goal is to use this knowledge to predict the binding affinity of the test sample.

### 4.2. Retrieval step

This step retrieves samples from the training dataset for each input query sample. Ground truth knowledge could help to retrieve samples during the training phase. However, this knowledge is not provided in the test phase. We have suggested three ways to retrieve samples. In the first approach, we could compute the distance between the feature descriptors of the test samples and all the training samples using a distance metric, such as the Euclidean distance metric, and then retrieve the closest ones. The disadvantage of this step is that if the similarity measure and feature descriptor fail to account for the distance between samples, this error is propagated to subsequent steps. The second approach is to retrieve samples randomly such that half of the retrieved samples belong to the active samples class and the other half belong to the inactive class. Half the samples were retrieved randomly. The third approach is to retrieve samples automatically. To clarify, the third approach involves a multi-step process. First, we compute the Euclidean distance between the query sample's feature descriptor and all positive samples (i.e., active samples) to measure similarity. Next, we rank the positive samples based on these similarity values. For the top half of the retrieved samples, we analyze each sample's neighborhood. If a sample has neighboring samples with conflicting labels, we flag it as a “hard sample” and include it in our retrieval set. These hard samples are particularly valuable because they tend to lie near the decision boundary, where model uncertainty is highest, making them critical for refining the model's discriminative ability.

The retrieval step identifies hard training samples for each query. Let $D=\{({𝐱}_{\text{i}},{\text{y}}_{\text{i}}){\}}_{\text{i}=\text{1}}^{\text{N}}$ denote the training set, where ${𝐱}_{\text{i}}\in R^{\text{256}}$ is the feature descriptor of protein-ligand pair i (learned by the feature encoder) and ${\text{y}}_{\text{i}}\in \text{R}$ is its continuous binding affinity. For binary classification, we define ${\text{z}}_{\text{i}}\in \{\text{0},\text{1}\}$ as the binary label obtained by thresholding ${\text{y}}_{\text{i}}$ (thresholds described in Section 4).

For a query q with feature descriptor ${𝐱}_{q}$, we compute the Euclidean distance to all training samples:


$$\begin{array}{c} d({𝐱}_{q},{𝐱}_{i})=∥{𝐱}_{q}-{𝐱}_{i}{∥}_{\text{2}} \end{array}$$ (2)

The k-nearest neighbor set is defined as:


$$\begin{array}{c} N_{k}(q)=\{i\in \{\text{1},\ldots ,N\}|d({𝐱}_{q},{𝐱}_{i})\text{ is among the }k\text{ smallest distances}\} \end{array}$$ (3)

In all experiments, we set k=10.

For practical comparison with existing methods, we also employ a binary version:


$$\begin{array}{c} i\text{ is hard if }i\in N_{k}(q)\text{ and }\exists j\in N_{k}(q)\text{ such that }z_{i}\neq z_{j} \end{array}$$ (4)

That is, a sample is hard if its binary label conflicts with at least one of its nearest neighbors, indicating it lies near the decision boundary.

The hard sample retrieval set is then:


$$\begin{array}{c} S_{\text{hard}}(q)=\{i\in N_{k}(q)|i\text{ satisfies the hard definition}\} \end{array}$$ (5)

The size of $S_{\text{hard}}$ is variable and depends on the local structure of the feature space around q.

We define hard samples based on the principle of manifold smoothness in feature space. The key assumption is that samples with similar molecular structures (and thus similar feature representations) should have similar binding affinity values. Hard samples are those that violate this assumption—they are close in feature space but have dissimilar outputs.

For practical purposes, we use a binary approximation where continuous affinity values are thresholded to obtain active/inactive labels. A training sample is considered hard if it is among the nearest neighbors of the query in feature space but has a different binary label than the query. This identifies samples that lie near the decision boundary in feature space—regions where the structure-affinity mapping is challenging and where model uncertainty is highest.

For samples that don't meet the hard sample criteria, we apply random dropout selection with a 50% probability to maintain diversity in our training set. Finally, we repeat this entire process for inactive samples to ensure balanced selection. This approach enables us to focus on challenging and informative examples while preventing the model from overlooking less obvious but still important cases.

Regarding the disadvantage of the first approach, we acknowledge that the similarity measure and feature descriptor may fail to account for the distance between samples, leading to errors in the retrieval process. The third approach overcomes this limitation in two ways:

- Robustness to noise: By using a neighborhood analysis, we can identify hard samples that are likely to be noisy or outliers, and add them to the retrieval set. This helps to reduce the impact of noise on the retrieval process.
- Diversity in retrieval: The dropout selection mechanism ensures that we have a diverse set of samples in the retrieval set, which helps to avoid bias in the results. By randomly selecting samples with a predetermined probability, we can explore new options and avoid overfitting to a specific subset of samples.

We believe that the third approach strikes a good balance between exploration and exploitation, thereby enhancing the model's efficiency. By focusing on hard samples near the decision boundary, we can improve the model's discriminative power and robustness. Meanwhile, the dropout selection mechanism helps maintain diversity and prevent overfitting.

The intuition behind the proposed retrieval approach is based on manifold smoothness; the local neighborhood around a given query point should belong to the same class label. If we could train a model that considers this concept, we could improve the model's generalization ability. We could train a more discriminative and generalizable model by ensuring local manifold smoothness around labeled and unlabeled data. In the proposed approach, this issue is considered by retrieving samples that do not satisfy the manifold smoothness and utilizing these samples in the task prediction step (through semi-supervised GCN).

### 4.3. Feature encoder

This study uses a convolutional neural network (CNN) to design a feature encoder for protein-ligand pairs. For this purpose, two 1D CNNs are considered for ligand and protein sequences (see Fig 1, feature extraction module). Let $F^{drug}\in R^{n_{\text{1}}\times k}$ and $F^{protein}\in R^{n_{\text{2}}\times k}$ denote the obtained feature vector of drug and protein CNNs, respectively, where $n_{\text{1}}$ and $n_{\text{2}}$ respectively show the length of the SMILES of a drug molecule and the length of the protein sequence. The drug feature extraction network $F^{drug}$ outputs a matrix where each row represents the feature vector of the corresponding SMILES token in the input sequence. Similarly, the protein feature extraction network $F^{protein}$ generates a matrix in which the row i encodes the feature vector of the $i^{th}$ amino acid in the input protein sequence*.* Then, the outputs of these CNNs are concatenated to produce the descriptor of the protein-ligand pair, which is shown by $F\in R^{n}$. In the proposed approach, the CNN architecture is designed similarly to [17].

### 4.4. Semi-supervised GCN

**Fig 2:**
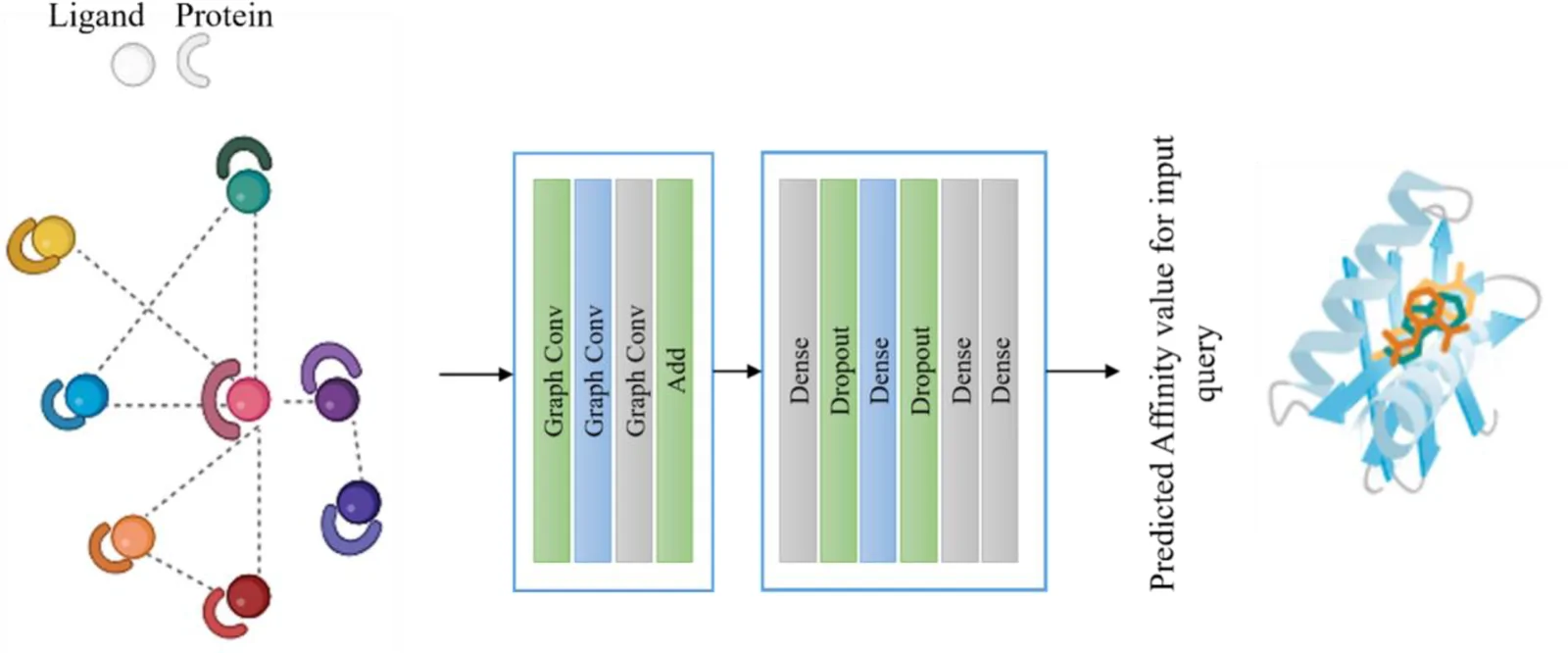
The proposed approach's overall schematic of the task predictor network. After constructing a graph using the query sample and retrieved samples, it is fed into the GCN to predict the affinity value of the query sample.

The prediction step is performed using a semi-supervised graph convolutional network. This network comprises three hidden graph convolutional layers (see Fig 2). The input and output of each graph convolutional layer are the graphs. Hence, we need to construct a graph to feed into the GCN. In the proposed approach, each graph node corresponds to a protein-ligand pair. The graph has |S|+1 nodes, where one of these is a test protein-ligand pair, and the remaining ones are the retrieved training protein-ligand pairs. Let *X* and *A* denote the node descriptor matrix and adjacency matrix of the input to GCN. Each row of the matrix *X* represents a node, a descriptor of a protein-ligand pair in our approach. The output of the feature encoder network is used to construct the node descriptor matrix *X*. The Adjacency matrix A is a square matrix where each matrix element indicates whether the corresponding protein-ligand pairs are adjacent or not. It should be noted that |S| out of |S|+1 nodes of the graph correspond to the training samples (retrieved samples for the query pair); therefore, we could utilize the label information of these samples to construct some part of the matrix A. Hence, |S|×|S| elements of matrix *A* are shown by $\hat{A}$. These elements are set to one if the corresponding nodes have similar labels. Otherwise, they are set to zero. The other elements (connections between test samples and |S| training samples) are learned during the training phase, which is explained in section 4.5.

A GCN processes the constructed graph to extract structural features, which are then passed through a Multilayer Perceptron (MLP) to predict protein-ligand binding affinity. For the regression task, we use a linear activation function in the final layer and optimize using mean squared error loss:


$$\begin{array}{c} L_{\text{1}}=\sum_{i=\text{1}}^{N}{\left\|y^{i}-o^{i}\right\|}^{\text{2}} \end{array}$$ (6)

where $o^{i}$ is the predicted label for the i^th^ sample. For binary classification of binding activity (active/inactive), we employ categorical cross-entropy loss.

### 4.5. Graph convolutional network

This section explains how a graph convolutional network works. The following describes the function of the graph convolutional layer. It takes *X* and *A* as input and then computes the output with *m* filters as follows:


$$\begin{array}{c} Z={a(\tilde{D}}^{-\frac{\text{1}}{\text{2}}}\tilde{A}{\tilde{D}}^{-\frac{\text{1}}{\text{2}}}X\theta ) \end{array}$$ (7)

where $\theta \in R^{n\times m}$ is a matrix of filter parameters, and a(.) is an activation function. The descriptions of matrices *X* and *A* are given in the previous section. Also, $\tilde{A}=A+I_{N}$, where $I_{N}$ is an N×N identity matrix. The elements of $\tilde{D}$ are defined as follows:


$$\begin{array}{c} {\tilde{D}}_{ii}=\sum_{j}{\tilde{A}}_{ij} \end{array}$$ (8)

In the case of the classification task, the activation function of the last layer is the sigmoid function. From a biological perspective, in our approach, when a graph passes through a graph convolution layer, the feature vectors of each node's corresponding neighboring nodes (similar protein-ligand pairs) are combined to form a new feature vector for the node. In these combinations, only similar samples are incorporated in learning a new feature vector for the protein-ligand pair. Hence, the adjacency matrix plays a crucial role because it defines the neighboring nodes. The loss function of this network is defined as follows:


$$\begin{array}{c} L_{\text{1}}=\sum_{i=\text{1}}^{N}\sum_{j=\text{1}}^{c}{\text{y}}_{j}^{i}\text{log}o_{j}^{i} \end{array}$$ (9)

Where *c* is the number of the class label, and $o^{i}$ denotes the output of the GCN for the *i*^th^ training protein-ligand pair.

### 4.6. Adjacency matrix

In this section, the adjacency of the input sample and |S| training samples are learned. To do so, the network $N^{adj}$ is designed. In the proposed approach, two samples are considered similar if they have the same label. Hence, we predict the label for the input sample and then use the predicted labels to compute the adjacency of the test sample and |S| training sample. To do so, the network $N^{adj}$ includes a self-attention layer and a multi-layer perceptron (MLP) (see Fig 1- learn adjacency matrix module). The attention layer computes the interaction of the input (feature vector) with itself to determine which part of the input should pay more attention to. The outputs are aggregations of these interactions and the attention scores. Then, the attention layer output is fed into the MLP, which contains multiple fully connected layers. The last layer is a fully connected layer with one neuron whose activation function is sigmoid.

The main goal of this layer is to predict the label of the input sample to utilize it to compute its adjacency with |S| retrieved training samples. They are similar if the input and retrieved training samples have the same label. Network $N^{adj}$ performs as a binary classifier: active or inactive. All training data is used to train this network. If the labels of samples are continuous, they should be converted to discrete through thresholding. Let $o^{test}$ denote the output of the network $N^{adj}$. In this case, the loss function of the network $N^{adj}$ is a binary cross-entropy, which is defined as follows:


$$\begin{array}{c} L_{\text{2}}=\sum_{i=\text{1}}^{\text{N}}y^{i}\text{log}o_{i}+(\text{1}-y^{i})\text{log}\left(\text{1}-o_{i}\right) \end{array}$$ (10)

where $y^{i}$ is the ground-truth label of the *i*^th^ protein-ligand pair. If $y^{i}$ is continuous, it is converted to a binary class (active/inactive) through thresholding. Also, $o_{i}$ denotes the output of the network.

Given an input query, which consists of a drug-protein pair shown by $\left(c^{input},p^{input}\right)$, we first pass it through the feature extractor network to obtain a set of extracted features. These features are then used as input to the $N^{adj}$ module, which predicts a pseudo label for the input pair as follows:


$$\begin{array}{c} o^{input}=N^{adj}\left(F\left(concate\left(F^{drug}\left(c^{input}\right),F^{protein}\left(p^{input}\right)\right)\right)\right) \end{array}$$ (11)

In parallel, we compute the set of retrieved samples that are most relevant to the input pair. To construct the graph that fed into the GCN, we need to define the adjacency matrix, which represents the connection knowledge between the input pair and the retrieved pairs as follows:


$$\begin{array}{c} \overset{ˇ}{A}=\text{repmat}\left(o^{input},|S|\right)\circ y+\text{repmat}\left({\text{1}-o}^{input},|S|\right)\circ (\text{1}-y) \end{array}$$ (12)

Where y is a vector with |S| dimension, which denotes the labels of the retrieved samples. Also, ∘ is the element-wise product operator. The repmat operator replicates an array ($o^{input}$) several times (in this formula, |S| times). By putting $\hat{A}$ and $\overset{ˇ}{A}$ together, the adjacency matrix *A* is created. The following preprocessing should be done to fed it as an adjacency matrix to the GCN layer:


$$\begin{array}{c} \tilde{A}=A+I_{N} \end{array}$$ (13)

$$\begin{array}{c} {\tilde{D}}_{ii}=\sum_{j}{\tilde{A}}_{ij} \end{array}$$ (14)

$$\begin{array}{c} {A=\tilde{D}}^{-\frac{\text{1}}{\text{2}}}\tilde{A}{\tilde{D}}^{-\frac{\text{1}}{\text{2}}} \end{array}$$ (15)

These formulas are used to normalize a graph's adjacency matrix, which is a common step in graph analysis and machine learning. The process involves modifying the matrix, calculating node degrees, and applying a normalization technique to scale the matrix. The resulting normalized matrix can be used in various applications, such as graph clustering, node embedding, and network analysis. The matrix *A* is finally fed into the semi-GCN as the adjacency matrix.

So far, we've established two key loss functions. The primary loss function, $L_{\text{1}}$, concentrates on optimizing the overall output of our proposed model. In contrast, the secondary loss function, $L_{\text{2}}$, targets the intermediate output, which plays a crucial role in forming the adjacency matrix. The overall loss function of the proposed approach is ${L_{\text{1}}+L}_{\text{2}}$. The proposed approach is learned end-to-end. To learn the proposed network, at first, the feature encoder network along with the adjacency network is trained using training data $D={\left\{\left(c^{i},p^{i}\right),y^{i}\right\}}_{i=\text{1}}^{N}$. Then, we learn a semi-supervised GCN using the constructed graph of the input sample and the retrieved training samples.

## 5. Method evaluation

In this section, experimental results are given. The proposed approach is evaluated on four well-known datasets in PLA, including PDBbind, Davis, KIBA, and BindingDB. A detailed description of these datasets is provided in Section 3.

To compare the proposed approach with the current state-of-the-art approaches, we choose KronRLS [45], SimBoost [36], DeepAffinity [44], DeepDTA [21], DeepCDA [17], SimCNN-DTA [28], GraphDTA [30], MSF-DTA [46], GEFormerDTA [47], TEFDTA [48], and NerLTR-DTA [27] as baseline approaches. In this case, the results of base approaches are taken directly from their original publications, except that we explicitly state that we train them.

In the proposed approach, the nested cross-validation approach is similar to [17,21,37], is used on the KIBA and Davis datasets. In this case, the dataset is randomly split into six equal parts, where five parts are chosen as the training set via 5-fold cross-validation, and the remaining part is used as the test set. It should be noted that the test set is entirely different from the training sets. The final results are the average of the five outcomes. In BindingDB, the training and test sets are obtained similarly to [17,44]. It should be noted that in whole approaches, we use the same split with comparable methods for a fair comparison.

This study evaluates the proposed approach in two ways: continuous binding affinity prediction (a regression task) and binary binding affinity prediction (a classification task). Since continuous binding affinity is provided in the four mentioned datasets, we use thresholds to convert it to binary (active or inactive) values. To this end, we have done something similar to [17,37]. The thresholds for the -log*k*_*d*_, KIBA, and -log*k*_*i*_ scores are 7, 12.1, and 7.6. In this case, all datasets, including both active and inactive samples, exhibit an uneven distribution. The unit of all measures, including *k*_*i*_, KIBA, and *k*_*d*_ is n*m*/*L*, where n*m* denotes nanomole. Note that *k*_*i*_ and *k*_*d*_ are transformed to the log space. For the PDBbind dataset, there is no predefined threshold to convert the continuous label to a discrete one. Hence, we get the median of the labels of the training samples and use it as the threshold.

For regression tasks, Concordance Index (CI), Mean Squared Error (MSE), $r_{m}^{\text{2}}$ index and the Pearson correlation coefficient (R) are used as evaluation metrics. CI investigates the concordance between the ground truth value and the predicted value. The intuition behind CI is that two protein-ligand pairs with different binding affinity values should be in the correct order. MSE is a well-known measure, and the index $r_{m}^{\text{2}}$ considers how the predictive value tends toward the mean. The Pearson correlation coefficient (R) indicates the linear correlation between the predicted and the ground truth values. If the predicted value and the ground truth value have a strong correlation, the Pearson correlation coefficient (R) gets a value of 1. Also, for the classification task, the Area Under the Precision-Recall curve (AUPR) is used as an evaluation measure [49]. As mentioned, in binary binding affinity, all datasets are imbalanced. Hence, AUPR is selected because it is suitable for situations with a significant skew in the class distribution.

The hyper-parameters optimization is done for the number of the filters (same for proteins and ligands) search over [16,32,64, 128, 256], the length of the filter size for ligands over [4,6,8,12], the length of the filter size for proteins over [8,12,16], and the learning rate for training network search over [0.1,0.01,0.001, 0.001,0.0005,0.0001,0.00005,0.00001]. The filter size for the protein sequence is larger than the SMILES sequence because the protein sequence length is greater than the length of the SMILES sequence. Additionally, to strike a balance between model performance and efficiency, we refrain from using large filter sizes. The results show that in most cases, eight for the ligand filter size and 12 for the protein filter size led to better performance. Also, 0.00001 is a more reliable learning rate for training the model. For the retrieval step, all the training samples are desired to be utilized in graph construction; however, this is computationally intractable.

This paper runs the program on a computer with an Intel(R) Core (T.M.) i9 CPU, NVIDIA GeForce GTX 1070 8 GB GDDR5, and 32 GB DDR4 RAM. We implemented our method using Python 3.8, TensorFlow, and Keras.

### 5.1. Ablation study

To evaluate the effectiveness of each module and key hyperparameters in the proposed method, we conducted ablation studies with two complementary analyses: First, we created three architectural versions. The first version, shown by $v_{\text{1}}$, we only utilized the drug and protein feature extractors to extract features from the input data. The concatenated features were then fed into a multi-layer perceptron (MLP) to predict the binding affinity. This version did not utilize semi-supervised GCN and is similar to the DeepDTA approach. The second version, shown by $v_{\text{2}}$, we used a first model to produce features for the drug and protein, which were then fed into a semi-supervised graph convolutional network (GCN). However, the learning of the drug and protein feature encoders was completely independent of the semi-supervised GCN, making this approach not end-to-end. The third version, called the full proposed model, includes the drug and protein feature extractors, as well as the semi-supervised GCN.

The results of the first ablation study on Davis are given in Table 1. As shown, the whole proposed model (the third version) achieves the best performance, outperforming the other two versions. This suggests that the knowledge retrieved from the samples and the end-to-end learning of the drug and protein feature encoders are essential components of the proposed method.

**Table 1:** Ablation study results on the Davis dataset evaluating the impact of different components of the proposed approach.

Approaches	CI ± std	MSE	$r_{m}^{2}$±std	AUPR±std
$v_{\text{1}}$	0.876 ± 0.005	0.263	0.648 ± 0.017	0.742 ± 0.010
$v_{\text{2}}$	0.906 ± 0.018	0.186	0.743 ± 0.014	0.781 ± 0.009
**RA-PLA**	**0.952 ± 0.005**	**0.145**	**0.801 ± 0.004**	**0.815 ± 0.005**

The results of the ablation study demonstrate the effectiveness of each module in the proposed method. The use of knowledge from retrieved samples and the end-to-end learning of the drug and protein feature encoders significantly improves the model's performance. The two-stage approach (the second version$v_{\text{2}}$) performs worse than the complete proposed model, indicating that the independent learning of the drug and protein feature encoders is less effective than the end-to-end learning approach. The version with only drug and protein feature extractors ($v_{\text{1}}$) performs the worst, highlighting the importance of utilizing the knowledge from the retrieved samples and the semi-supervised GCN in the proposed method.

Second, we systematically investigated the impact of retrieval set size on model performance, as this hyperparameter critically balances two key objectives: exploring diverse samples through random selection and exploiting informative cases through hard sample selection. We evaluated various retrieval set sizes {50, 100, 150, 200, 300} and found optimal performance at 150 samples. The results obtained are presented in Table 2. As shown, when the number of retrieved samples is set to 150, the proposed approach achieves better performance. Through extensive empirical validation, we observed that smaller retrieval sets may limit the model's exposure to challenging cases, while giant sets can dilute the importance of informative hard samples. Our chosen threshold of 0.50 for random selection – applied to both positive and negative samples- was carefully determined through systematic experimentation to optimize this trade-off.

**Table 2:** Performance comparison using different retrieval set sizes (50, 100, 150, 200, 300) on the Davis dataset.

Dataset	# of retrieved samples	CI ± std	R	MSE	$r_{m}^{2}$±std	AUPR±std
Davis	50	0.926 ± 0.004	0.887	0.146	0.792 ± 0.003	0.799 ± 0.001
	100	0.943 ± 0.010	0.896	0.165	0.791 ± 0.006	0.803 ± 0.001
	150	**0.952 ± 0.005**	**0.914**	**0.145**	**0.801 ± 0.004**	**0.815 ± 0.005**
	200	0.926 ± 0.007	0.891	0.198	0.785 ± 0.003	0.798 ± 0.008
	300	0.897 ± 0.002	0.864	0.217	0.764 ± 0.004	0.785 ± 0.001

Third, to justify our selection of the third retrieval strategy (hard sample retrieval with dropout), we evaluated all three strategies described in Section 4.2 on the Davis dataset. The results are presented in Table 3.

**Table 3:** Comparison of retrieval strategies on Davis dataset.

Dataset	# of retrieved samples	CI ± std	R	MSE	$r_{m}^{2}$±std	AUPR±std
Davis	Strategy 1 (Distance-based)	0.918 ± 0.006	0.884	0.182	0.768 ± 0.005	0.786 ± 0.007
	Strategy 2 (Random balanced)	0.881 ± 0.04	0.852	0.215	0.702 ± 0.006	0.731 ± 0.005
	Strategy 3 (Hard sample + dropout)	0.952 ± 0.005	0.914	0.145	0.801 ± 0.004	0.815 ± 0.005

- Strategy 1 (Distance-based): Retrieved the k-nearest training samples based on Euclidean distance in the feature space.
- Strategy 2 (Random balanced): Retrieved equal numbers of active and inactive samples randomly.
- Strategy 3 (Hard sample + dropout): Retrieved hard samples (those with conflicting neighbor labels) combined with 50% random dropout selection.

As shown in Table 3, the third strategy consistently outperforms both alternatives across all metrics. Strategy 1 suffers from propagation of errors in similarity measurement and feature representation, while Strategy 2 lacks the informative hard samples that lie near decision boundaries. Strategy 3 strikes the optimal balance between exploration (diverse samples) and exploitation (challenging, informative cases).

To evaluate the contribution of our label-dependent adjacency construction, we compared it with a standard k-NN (label-free) adjacency strategy. In the k-NN strategy, edges are established based solely on feature distance with equal weights, while our approach uses feature distance pseudo-labels for edge existence.

As shown in Table 4, our approach significantly outperforms *k*-NN (*k* is set to 3) across all metrics (CI: 0.952 vs. 0.918, p < 0.01). The improvement is particularly notable for AUPR (0.815 vs. 0.786), suggesting that label-dependent edge refinement is especially beneficial for distinguishing active from inactive compounds.

**Table 4:** Comparison of k-NN (label-free) adjacency and our proposed label-dependent adjacency strategy on the Davis dataset.

Strategy	CI ± std	R	MSE	r_m² ± std	AUPR±std
k-NN (label-free)	0.918 ± 0.006	0.884	0.182	0.768 ± 0.005	0.786 ± 0.007
RA-PLA (label-dependent)	0.952 ± 0.005	0.914	0.145	0.801 ± 0.004	0.815 ± 0.005

These results confirm that incorporating label information into adjacency construction—when properly constrained by feature-based connectivity—provides meaningful improvements over purely feature-based adjacency.

The comparison with *k*-NN adjacency (Table 4) demonstrates that our approach substantially outperforms label-free feature-based adjacency (CI: 0.952 vs. 0.918), confirming that the improvement comes from meaningful graph refinement rather than overfitting to label patterns.

### 5.2. Results

This section compares results on the PDBbind, Davis, KIBA, and BindingDB datasets with the base approaches.

For a fair comparison, the comparison should be conducted under the same setting as the state-of-the-art approaches. In PDBbind, two different settings are employed in state-of-the-art techniques. The first setting is the refined set as the core set (285 pairs), and the remaining samples are used as training samples. The results obtained in this setting are presented in Table 5. In this case, the proposed approach improves the Pearson correlation coefficient by 7.3% over the 1D2D-CNN approach.

**Table 5:** Comparing the proposed approach results with base approaches on the PDBbind Dataset (the first setting).

Approaches	R	RMSE
1D2D-CNN [50]	0.848	1.64
DCML [32]	0.843	1.255
**RA-PLA**	**0.921**	**0.945**

In the second setting, PDBbind is divided randomly into six parts, where one part is designated as the test set, and the remaining five parts are used to train the model using five-fold cross-validation. The results obtained using the PDBbind dataset in the second setting are presented in Table 6. As shown, the proposed approach outperforms the other approaches significantly. It improves the CI measure by 11.4% compared to the PLA-MoRe approach. Also, in MSE, our approach gets the lowest value compared to the other methods. One of the main contributions that led to better performance is that the knowledge of the retrieved samples is also considered in the task prediction. Based on the retrieval algorithm, all hard samples, neighboring samples near the query sample but with different labels, are incorporated to infer the label of the query sample. The class labels refer to the active/inactive classes.

**Table 6:** Comparing the proposed approach results with base approaches on the PDBbind Dataset (the second setting).

Approaches	CI ± std	MSE	RMSE	$r_{m}^{2}$±std
SimBoost ^36^	0.74 ± 0.005	1.901 ± 0.023	1.378 ± 0.008	**0.621 ± 0.014**
DeepDTA [21]	0.745 ± 0.002	1.810 ± 0.003	1.346 ± 0.013	**0.639 ± 0.021**
GraphDTA [30]	0.734 ± 0.007	1.993 ± 0.053	1.411 ± 0.018	**0.636 ± 0.014**
PLA-MoRe [34]	0.780 ± 0.004	1.515 ± 0.032	1.230 ± 0.013	**0.619 ± 0.017**
**RA-PLA**	**0.894 ± 0.002**	**0.961 ± 0.025**	**0.980 ± 0.013**	**0.674 ± 0.017**

Regarding the datasets in which the corresponding label for each protein-ligand pair is a continuous value, we should convert the continuous value to a binary value using thresholding. However, there is no predefined threshold for the PDBbind dataset to convert the continuous label to a discrete one. Hence, we get the median of the labels of the training samples and use it as the threshold. It should be noted that the retrieved hard samples serve as support vectors in SVM, aiding the model in learning more accurate decision boundaries for the query sample.

Also, in this paper, for evaluation, we use three external test sets: the PDBbind 2013 core set, the PDBbind 2016 core set, and the PDBbind 2019 holdout set. The 2019 holdout set simulates a temporal split scenario, where a model trained on prior structures is used to predict binding affinities for later released structures. Notably, there is no overlap among training, validation, and testing sets.

To ensure fair comparison and to provide a comprehensive evaluation across all standard test versions, we report results on all three test set versions in Table 7. Our training set is consistent with the protocol in [32], which has been widely adopted for evaluating structure-based affinity prediction methods.

**Table 7:** Comparative evaluation of RA-PLA on three standard PDBbind test sets (2013 core set, 2016 core set, and 2019 holdout set).

Approaches	CI	MSE	RMSE	$r_{m}^{2}$
**PDBbind 2013 holdout set**	**0.885**	**1.019**	**1.009**	**0.681**
**PDBbind 2016 holdout set**	**0.894**	**0.961**	**0.980**	**0.674**
**PDBbind 2019 holdout set**	**0.812**	**1.456**	**1.206**	**0.619**

The comparative evaluation of RA-PLA across the three PDBbind test sets reveals a clear and expected performance gradient, with the model achieving its strongest results on the 2013 and 2016 core sets and a notable decline on the more challenging 2019 holdout set. Specifically, the concordance index (CI) remains robust for the 2013 and 2016 sets at 0.885 and 0.894, respectively, alongside low mean squared errors (MSE) of 1.019 and 0.961 and high squared correlation coefficients ($r_{m}^{\text{2}}$) of 0.681 and 0.674, indicating reliable predictive power and good generalizability for these earlier benchmarks. However, performance drops significantly on the 2019 set, with the CI falling to 0.812, the MSE nearly doubling to 1.456, and the $r_{m}^{\text{2}}$ decreasing to 0.619, which suggests that RA-PLA struggles with the greater chemical and structural diversity or the more difficult binding affinity cases present in the newer data. This degradation highlights a key limitation in the model's ability to extrapolate to unseen, more heterogeneous datasets, underscoring the need for enhanced feature representation or transfer learning strategies to bridge the performance gap between historical and contemporary benchmarks.

In Table 8, the results obtained from the Davis dataset are presented and compared with the base approaches. The proposed method performs better in all metrics: the CI measure, R, MSE, $r_{m}^{\text{2}}$ and AUPR. Utilizing similar training samples in task prediction steps helps the approach achieve better results. Our approach improves the CI measure by 1.6% compared to the other comparable approaches. Additionally, it demonstrates a 7.6% improvement in AUPR compared to the base approaches. It should be noted that the boldfaced values indicate that the corresponding method outperforms the comparison methods.

**Table 8:** Comparing the proposed approach results with base approaches on the Davis, KIBA, and BindingDB Datasets.

Davis dataset						Paired t-test
Approaches	CI ± std	R	MSE	$r_{m}^{2}$±std	AUPR±std	p-value
KronRLS [45]	0.871 ± 0.0008	–	0.379	0.407 ± 0.005	0.661 ± 0.010	1.56 × 10^−1^
SimBoost [37]	0.872 ± 0.002	–	0.282	0.644 ± 0.006	0.709 ± 0.008	3.69 × 10^−2^
DeepDTA [21]	0.878 ± 0.004	0.846	0.261	0.630 ± 0.017	0.714 ± 0.010	2.19 × 10^−2^
DeepCDA [17]	0.891 ± 0.003	0.857	0.248	0.649 ± 0.009	0.739 ± 0.006	3.00 × 10^−2^
SimCNN-DTA [28]	0.855 ± 0.0022	–	0.305	0.5952 ± 0.0138	0.657 ± 0.0076	3.95 × 10^−2^
GraphDTA [30]	0.893	–	0.229	–	–	–
NerLTR-DTA [27]	0.936	–	0.155	0.792	–	–
G* + protein similarity [48]	0.87	–	0.26	–	–	–
MSF-DTA [46]	0.906	–	0.194	–	–	–
GEFormerDTA [47]	0.895	–	0.212	0.735	–	–
TEFDTA [48]	0.890 ± 0.002	–	0.199	0.756 ± 0.008	–	–
DeepPS [52]	–	–	–	0.546 ± 0.003	0.710 ± 0.003	–
GDilatedDTA [53]	0.885	–	0.237	0.686	–	
DeepDTAGen [54]	0.890 ± 0.004	–	0.214 ± 0.006	0.705 ± 0.001	0.772 ± 0.002	
BTDHDTA [55]	0.907	0.733	0.201			
MGF-DTA [56]	0.913	0.779	0.183	–	–	–
**RA-PLA**	**0.952 ± 0.005**	**0.914**	**0.145**	**0.801 ± 0.004**	**0.815 ± 0.005**	
**KIBA dataset**						
KronRLS [45]	0.782 ± 0.0009	–	0.411	0.342 ± 0.001	0.635 ± 0.004	9.87 × 10^−2^
SimBoost [36]	0.836 ± 0.001	–	0.222	0.629 ± 0.007	0.760 ± 0.003	3.88 × 10^−2^
DeepDTA [21]	0.863 ± 0.002	0.848	0.194	0.673 ± 0.009	0.788 ± 0.004	1.97 × 10^−2^
DeepCDA [17]	0.889 ± 0.002	0.855	0.176	0.682 ± 0.008	0.812 ± 0.005	4.80 × 10^−2^
SimCNN-DTA [28]	0.821 ± 0.0011	–	0.257	0.5734 ± 0.0034	0.721 ± 0.0018	4.90 × 10^−2^
GraphDTA [30]	0.891	–	0.139	–	**–**	**–**
NerLTR-DTA [27]	0.893	–	0.134	0.800	**–**	**–**
G* + protein similarity [48]	0.80	**–**	0.29	**–**	**–**	**–**
MSF-DTA [46]	0.899	**–**	0.124	**–**	**–**	**–**
GEFormerDTA [47]	0.877		**0.081**	**0.844**	**–**	**–**
TEFDTA [48]	0.860 ± 0.001	**–**	0.184	0.731 ± 0.006	**–**	**–**
DeepPS [52]	–	–	–	0.604 ± 0.003	0.762 ± 0.004	–
GDilatedDTA [53]	0.876	–	0.156	0.775	–	–
DeepDTAGen [54]	0.897 ± 0.003		0.146 ± 0.008	0.765 ± 0.005	0.843 ± 0.001	–
BTDHDTA [55]	0.898	0.805	0.127	–	–	–
MGF-DTA [56]	0.897	**0.897**	0.132	–	–	–
RA-PLA	**0.916 ± 0.002**	0.894	0.124	0.791 ± 0.002	**0.871 ± 0.005**	
**BindingDB dataset**						
DeepDTA [21]	0.812 ± 0.02	0.832	0.824	0.623 ± 0.02	0.443 ± 0.01	3.09 × 10^−3^
DeepCDA [17]	0.822 ± 0.01	0.844	0.808	0.631 ± 0.02	0.459 ± 0.03	2.71 × 10^−3^
DeepAffinity [44]	–	0.81	0.91	–	–	–
[51]	**0.927**	–	–	0.365	–	–
GDilatedDTA (RT) [53]	0.868 ± 0.001		0.483 ± 0.002	0.730 ± 0.001	0.820 ± 0.00021	
DeepDTAGen [54]	0.876 ± 0.004		0.458 ± 0.002	0.760 ± 0.003	0.870 ± 0.004	
RA-PLA	0.862 ± 0.006	**0.883**	**0.701**	**0.671 ± 0.02**	**0.518 ± 0.009**	

*Note: “-” means that they are not reported in the original report.

Table 7 also shows the achieved results of the proposed approach on the KIBA dataset. Also, it is compared with the results of the base approaches. In most measures, the proposed method provides the best results. It achieves a 5.9% improvement in AUPR over the second-best (DeepCDA). Our approach improves the CI measure by 1.7% compared to the other comparable approaches.

The results of our approach, along with its comparison to base approaches on the BindingDB dataset, are also presented in Table 8. Our approach yields the best results in this case across four measures. It shows a 5.9% improvement in AUPR. It should be noted that [51] utilizes smaller training and test sets. Their training set consists of 19312 samples, and the test set comprises 13289 samples. Our method and the other comparison methods in Table 7 use the same training set (with 101,134 samples) and test set (with 43,391 samples).

To statistically verify the proposed method, we have used the paired t-test. In this test, the null hypothesis ($H_{\text{0}}$) states that there is no difference between the proposed method and the comparison methods, and the alternative hypothesis ($H_{\text{1}}$) shows significant differences between them. As shown in the KIBA and BindingDB datasets, all reported p-values are below 0.05, indicating that we reject the null hypothesis and demonstrate a statistically significant difference between the proposed and comparable methods. It should be noted that to compute a reliable p-value, we require at least three values. This is the reason why we do not report the p-value for some approaches. Additionally, in all reported p-values, the MSE metric is not used because, in all metrics except MSE, a higher value is considered better.

### 5.3. Generalizability

In this section, the generalizability of the proposed approach is evaluated and applied to the BindingDB dataset. As mentioned in this dataset, four groups of proteins are entirely excluded from the training and test sets: nuclear estrogen receptors (516 samples), ion channels (8,101 samples), receptor tyrosine kinases (3,355 samples), and G-protein-coupled receptors (77,994 samples). As a result, an experiment is designed to evaluate how the proposed approach performs when a target protein is not seen during the training phase. The model is trained on a BindingDB dataset and then applied to nuclear estrogen receptors, ion channels, receptor tyrosine kinases, and G-protein-coupled receptors.

The results obtained from the other approaches are compared in Table 9. It shows that our approach in all datasets performs best in the CI, R, $r_{m}^{\text{2}}$ and AUPR measures compared to the other base approaches. Moreover, the proposed approach achieves an average improvement of 15.5% in AUPR. These results demonstrate that the proposed approach outperforms other methods in the classification task. Additionally, in the MSE metric, our approach outperforms DeepAffinity in three out of four datasets. For ER and Tyrosine kinase, all p-values are below 0.05, indicating that the proposed method outperforms DeepDTA and DeepCDA. Additionally, for the Ion channel and GPCR, all p-values are less than 0.08, suggesting that there's less than an 8% chance that the proposed method and the comparison methods are not different.

**Table 9:** Generalizability evaluation of our approach and base comparable approaches on BindingDB. In this case, the model is learned on the BindingDB dataset and evaluated on four other datasets.

Dataset	Metrics	Approaches			
		DeepAffinity [44]	DeepDTA [21]	DeepCDA [17]	RA-PLA
ER	CI	–	0.50	0.53	**0.63**
	R	0.09	0.004	0.10	**0.19**
	MSE	1.76	3.73	3.85	**1.62**
	$r_{m}^{\text{2}}$	–	0.006	0.004	**0.24**
	AUPR	–	0.10	0.12	**0.30**
Paired t-test	p-value	–	3.24 × 10^−3^	2.20 × 10^−2^	
Ion channel	CI	–	0.60	0.60	**0.71**
	R	0.23	**0.31**	**0.31**	**0.31**
	MSE	1.79	2.78	2.50	**1.48**
	$r_{m}^{\text{2}}$	–	0.05	0.08	**0.15**
	AUPR	–	0.20	0.22	**0.35**
Paired t-test	p-value	–	6.65 × 10^−2^	7.36 × 10^−2^	
GPCR	CI	–	0.59	0.60	**0.72**
	R	0.21	0.28	0.28	**0.32**
	MSE	**1.50**	2.15	2.67	1.63
	$r_{m}^{\text{2}}$	–	0.05	0.06	**0.12**
	AUPR	–	0.13	0.15	**0.34**
Paired t-test	p-value	–	5.76 × 10^−2^	5.60 × 10^−2^	
Tyrosine kinase	CI	–	0.65	0.67	**0.69**
	R	0.16	0.36	0.42	**0.51**
	MSE	2.10	4.08	3.14	**2.04**
	$r_{m}^{\text{2}}$	–	0.09	0.13	**0.28**
	AUPR	–	0.17	0.19	**0.31**
Paired t-test	p-value	–	2.66 × 10^−2^	2.20 × 10^−2^	

*Note: “-” means that they are not reported in the original report.

### 5.4. Target selectivity of ligands

To evaluate the proposed approach, we have done an experiment that assesses the target selectivity of ligands. In this experiment, we have a pool of ligands. Then, for each target, we compute the interaction affinity of the given target with a pool of ligand molecules to demonstrate how the proposed approach can predict the true order of interactions. We have chosen the Protein-tyrosine phosphatases (PTPs) family as protein targets. These protein families are involved in metabolic regulation and mitogenic signal transduction processes. The selected proteins in the PTP family are SHP1, PTPRC, PTPRE, PTPRA, and PTP1B. Also, we have used the following three ligands:

1) 2-(oxaloamino) benzoic acid with PubChem CID of 444764,
2) 3-(carboxyformamido) thiophene-2-carboxylic acid with PubChem CID of 44359299 and
3) 2-formamido-4,5,6,7-tetrahydrothieno [2,3-c]pyridine-3-carboxylic acid with PubChem CID of 90765696.

**Fig 3:**
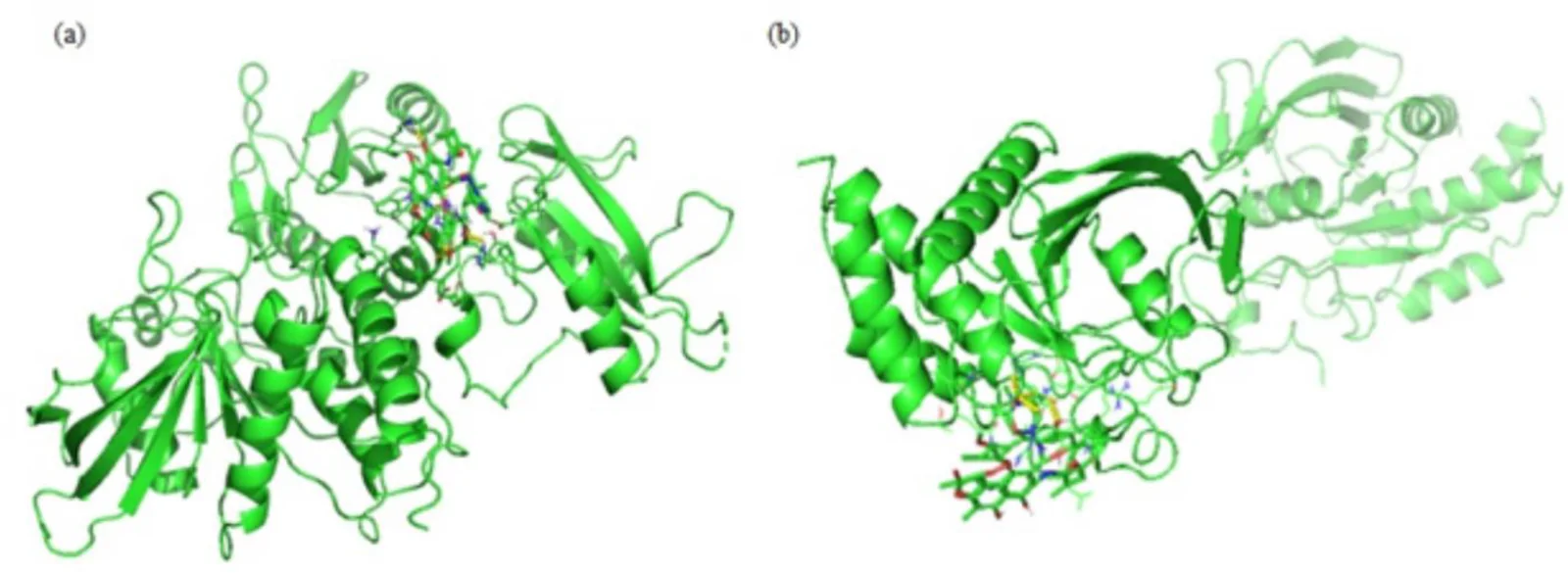
The predicted ligand-protein complex. (a) PubChem CID of 90765696 drug and SHP1 protein and (b) PubChem CID of 90765696 drug and PTPRA protein. AutoDock Vina does this prediction.

Recent studies show that all selected ligands are more attracted to the PTP1B protein. The $pK_{i}$ values for the three ligands against PTP1B, respectively, are 4.63, 4.25, and 6.69. Also, the difference between $pK_{i}$ value against PTP1B and the second closest protein (shown by $\Delta pK_{i}$) respectively are 0.75, 0.7, and 2.47. The results obtained are presented in Table 10. Our approach predicts the affinity value in the same order as the ground truth one. Also, for the third compound, the predicted $\Delta pK_{i}$ for our approach is closer to the true value compared with the other methods. To further evaluate the results, we have done molecular docking between the third ligand and the proteins that get the second-highest $pK_{i}$ value against the third ligand in RA-PLA and DeepCDA approaches. In other words, molecular docking is done between two pairs: 1) molecular docking between the ligand with PubChem CID of 90765696 and the SHP1 protein, and 2) molecular docking between the ligand with PubChem CID of 90765696 and the PTPRA protein. To achieve this, we utilized AutoDock Vina. The docking scores reported in Table 11 represent calculated binding energies from AutoDock Vina (in kcal/mol), which serve as a computational estimate of binding strength. Lower (more negative) binding energies indicate stronger predicted binding, which is consistent with the ranking order predicted by RA-PLA and validated by AutoDock Vina's independent scoring. RA-PLA and AutoDock Vina predict the same order for the two pairs mentioned, while the predicted orders of DeepCDA and DeepDTA do not match those predicted by AutoDock Vina. Ligand efficiency is another metric reported, which expresses the binding energy per atom of the ligand to the protein. Also, the predicted ligand-protein complex for these two pairs is shown in Fig 3.

**Table 10:** The predicted value for compounds with PubChem CIDs of 444764 (C1), 44359299 (C2), and 90765696 (C3) against five proteins of the PTP family.

proteins	DeepDTA			DeepCDA			RA-PLA		
	C1	C2	C3	C1	C2	C3	C1	C2	C3
PTP1B	5.49	5.79	**5.84**	5.61	**5.93**	**6.12**	**5.89**	5.27	**6.47**
PTPRA	5.70	5.60	5.54	**5.70**	5.13	5.93	5.73	5.41	4.74
PTPRE	**5.86**	**5.89**	5.78	5.23	5.62	5.01	5.21	**6.12**	5.29
PTPRC	5.66	5.39	5.74	4.99	5.37	5.28	5.00	4.98	4.68
SHP1	5.72	5.67	5.50	5.35	5.46	5.69	4.66	5.23	5.42

**Table 11:** Molecular docking results between the PubChem CID of 90765696 drug and two proteins, SHP1 and PTPRA.

Drug CID	Target	Approach	Predicted $pK_{i}$	Results of molecular docking	
				Docking scores from AutoDock Vina	Ligand efficiency
90765696	SHP1	RA-PLA	5.42	4.40 (- 0.64)	−0.19
90765696	SHP1	DeepCDA	5.69	4.40 (- 0.64)	−0.19
90765696	SHP1	DeepDTA	5.50	4.40 (- 0.64)	−0.19
90765696	PTPRA	RA-PLA	4.74	10.24 (- 1.01)	−0.18
90765696	PTPRA	DeepCDA	5.93	10.24 (- 1.01)	−0.18
90765696	PTPRA	DeepDTA	5.54	10.24 (- 1.01)	−0.18

### 5.5. Screening power

In this section, the screening power of the proposed method is assessed. The screening power test evaluates the capability of the scoring function in discriminating true binders from decoy compounds. This test is conducted on the PDBBind2016-core set (also known as the CASF-2016 dataset). It contains 285 high-quality protein-ligand complexes with 57 clusters of complexes such that in each cluster, five ligands are considered active targets, and the remaining ones (i.e., 280 ligands) are considered inactive. In the PDBBind2016-core set, two settings for screening power tests are designed: the forward screening power test and the reverse screening power test. In the forward screening test, a potential compound is identified for a specific target, and in the reverse screening test, a likely target is determined for a particular active compound. In this section, we conducted the forward screening test, and the results are reported in Table 12. To report the results, we utilized the Enrichment Factor (EF) as an evaluation measure, similar to state-of-the-art approaches. EF measure is defined as follows:

**Table 12:** Screening power results on PDBBind2016-core set (CASF-2016 dataset).

Approach	${EF}_{1\%}$	${EF}_{5\%}$	${EF}_{10\%}$
ChemScore@GOLD	8.65	3.95	2.92
Autodock Vina	7.70	4.01	2.87
Δ_vina_RF_20_	11.73	4.43	3.10
Δ-AEScore	6.16	3.76	2.48
RA-PLA	8.15	4.68	2.98


$$\begin{array}{c} {EF}_{x\%}=\frac{{NTB}_{x\%}}{{NTB}_{total}\times x} \end{array}$$ (16)

where ${NTB}_{x\%}$denotes the number of true binders among the x% top-ranked candidates and ${NTB}_{total}$ refers to the number of true binders among all candidates.

The introduced scoring functions are divided into two categories: classical scoring functions and machine learning (ML) based scoring functions. The enrichment factor for ML-based scoring functions is, on average, lower than that of classical approaches [57]. In Table 12, we have compared the proposed method with ChemScore@GOLD (Special implementation of [58,59]), Autodock Vina [60], Δ_vina_RF_20_ [61], and Δ-AEScore [62]. Our approach gets results comparable to those of the classical methods. Δ_vina_RF_20_ has obtained a better result compared to the other approaches. However, it is shown that in this approach, there is a 50% overlap between protein-ligand complexes of the training and test sets [57]. In the proposed approach, we use only the SMILES representation of the drug and the sequence of amino acids of the protein as input. In other words, we do not use the 3D complex of ligand-protein or the protein's 3D structure.

## 6. Discussion

Predicting protein-ligand interactions is crucial for new drug discovery and repurposing. A binding affinity measures how strong a protein-ligand interaction is. Biologists can reduce the time spent on wet laboratory experiments and accelerate drug discovery by computationally predicting the binding affinity between ligands and proteins [27,63–65]. This paper proposes an end-to-end deep learning-based approach for PLA prediction that utilizes the knowledge of hard samples of protein-ligand pairs in the affinity prediction step. One of the main advantages of the proposed method is that it leverages the observation that a single model has limited ability to fit an extensive feature space (in this case, the protein-ligand feature space). To address this issue, the proposed approach retrieves query-dependent hard training samples and incorporates them into inferring the label of the query sample. The proposed method first retrieves a hard sample of protein-ligand pairs for the input sample. Then, it is fed into the network to compute the features for the input protein-ligand pair and the retrieved pairs. Next, these descriptors are fed into the network $N^{adj}$to compute the adjacency matrix. Then, a graph is constructed using node descriptors and an adjacency matrix in which each node represents a protein-ligand pair. This graph is fed into a semi-supervised GCN to predict the label of the node corresponding to the query protein-ligand pair. Learning of the proposed network is done end-to-end. The proposed approach uses GCN not as a feature descriptor but as a node classification model.

The evaluation results of the proposed approach demonstrate its successful performance on the PDBbind, Davis, KIBA, and BindingDB datasets. One of the findings of the proposed approach is that it learns a model with more generalizability. The learned model on BindingDB is applied to four other datasets where their target proteins are not seen in the training phase (cold target setting). In other words, the mentioned target proteins are entirely excluded from BindingDB. The results confirm that our approach has more generalization ability than other approaches. Additionally, the proposed approach achieves better classification performance than regression.

One of the key motivations for this paper is that current approaches have limited generalizability due to relying on a single learned model to predict affinity values for all unseen protein-ligand pairs. We believe that the generalizability of this approach is limited. To consider this issue, we have designed a method that utilizes hard-retrieved samples in the task prediction step. To this end, a semi-supervised GCN is used. In this case, in the inference step, for an input sample, a graph is constructed in which most nodes (retrieved protein-ligand pairs) have labels, and the label of the input sample is unknown. This graph is fed into the semi-supervised GCN, and training is then performed. Finally, the label of the input samples is predicted by utilizing the knowledge of hard samples. The training step adjusts the parameter values correctly, and in the inference step, the learned model is fine-tuned for the input sample. This leads to the advantage of being partly independent of the dataset, as fine-tuning for each input sample is performed during the inference step. The results obtained from the BindingDB dataset confirm this statement.

One of the key reasons the proposed method leads to an improvement in performance is that, in the inference step, it not only utilizes the learned model from the training data but also fine-tunes the model with retrieved similar training samples for the test sample, resulting in a more accurate prediction. It means that in the inference step, we extract similar training samples for the test sample and construct a semi-supervised graph. Next, the model is fine-tuned using the constructed semi-supervised graph. Then, a prediction is made for the test sample. It is worth noting that we have a specific model for each test sample.

One of the other advantages of the proposed approach that helps it learn a more discriminative model is the retrieval step. In the retrieval step, the number of retrieved samples is different for each query input. If the query sample is placed near the decision boundary, the number of hard samples increases, and all these samples are incorporated into the decision-making process. In the training step, the number of retrieved samples is initially large and decreases as the iterations progress. The reason is that during the training process, the model becomes progressively more accurate and discriminative.

The proposed approach could help biologists deal with biological problems. In this case, we have designed a screening power experiment on the PDBBindv2016-core set (i.e., CASF 2016). This experiment evaluates the capability of the proposed method in discriminating true binders from decoy compounds. The results show that it achieves a comparable ranking even with the classical scoring function, which utilizes much of the information from the 3D complex of the protein-ligand complex.

One of the limitations of the proposed method is that the inference step for each test sample is slower than the other techniques. Because, for each test sample, the model should be fine-tuned using the retrieved training samples. However, we believe that achieving better performance is a higher priority than minimizing inference time in PLA prediction. Also, the maximum number of retrieved samples for each query should be limited due to memory limitations.
